# Evidence and User Considerations of Home Health Monitoring for Older Adults: Scoping Review

**DOI:** 10.2196/40079

**Published:** 2022-11-28

**Authors:** Andrew Chan, Rachel Cohen, Katherine-Marie Robinson, Devvrat Bhardwaj, Geoffrey Gregson, Jeffrey W Jutai, Jason Millar, Adriana Ríos Rincón, Atena Roshan Fekr

**Affiliations:** 1 Faculty of Rehabilitation Medicine Department of Occupational Therapy University of Alberta Edmonton, AB Canada; 2 Innovation and Technology Hub Glenrose Rehabilitation Research Edmonton, AB Canada; 3 KITE Research Institute Toronto Rehabilitation Institute University Health Network Toronto, ON Canada; 4 Institute of Biomedical Engineering University of Toronto Toronto, ON Canada; 5 School of Engineering Design and Teaching Innovation Faculty of Engineering University of Ottawa Ottawa, ON Canada; 6 Department of Philosophy Faculty of Arts University of Ottawa Ottawa, ON Canada; 7 Department of Electrical Engineering and Computer Science Faculty of Engineering University of Ottawa Ottawa, ON Canada; 8 Interdisciplinary School of Health Sciences Faculty of Health Sciences University of Ottawa Ottawa, ON Canada; 9 LIFE Research Institute University of Ottawa Ottawa, ON Canada

**Keywords:** smart homes, concerns, user-centered frameworks, clinical evidence, home health monitoring, gerontechnology, telemonitoring, older adults

## Abstract

**Background:**

Home health monitoring shows promise in improving health outcomes; however, navigating the literature remains challenging given the breadth of evidence. There is a need to summarize the effectiveness of monitoring across health domains and identify gaps in the literature. In addition, ethical and user-centered frameworks are important to maximize the acceptability of health monitoring technologies.

**Objective:**

This review aimed to summarize the clinical evidence on home-based health monitoring through a scoping review and outline ethical and user concerns and discuss the challenges of the current user-oriented conceptual frameworks.

**Methods:**

A total of 2 literature reviews were conducted. We conducted a scoping review of systematic reviews in Scopus, MEDLINE, Embase, and CINAHL in July 2021. We included reviews examining the effectiveness of home-based health monitoring in older adults. The exclusion criteria included reviews with no clinical outcomes and lack of monitoring interventions (mobile health, telephone, video interventions, virtual reality, and robots). We conducted a quality assessment using the Assessment of Multiple Systematic Reviews (AMSTAR-2). We organized the outcomes by disease and summarized the type of outcomes as positive, inconclusive, or negative. Second, we conducted a literature review including both systematic reviews and original articles to identify ethical concerns and user-centered frameworks for smart home technology. The search was halted after saturation of the basic themes presented.

**Results:**

The scoping review found 822 systematic reviews, of which 94 (11%) were included and of those, 23 (24%) were of medium or high quality. Of these 23 studies, monitoring for heart failure or chronic obstructive pulmonary disease reduced exacerbations (4/7, 57%) and hospitalizations (5/6, 83%); improved hemoglobin A1c (1/2, 50%); improved safety for older adults at home and detected changing cognitive status (2/3, 66%) reviews; and improved physical activity, motor control in stroke, and pain in arthritis in (3/3, 100%) rehabilitation studies. The second literature review on ethics and user-centered frameworks found 19 papers focused on ethical concerns, with privacy (12/19, 63%), autonomy (12/19, 63%), and control (10/19, 53%) being the most common. An additional 7 user-centered frameworks were studied.

**Conclusions:**

Home health monitoring can improve health outcomes in heart failure, chronic obstructive pulmonary disease, and diabetes and increase physical activity, although review quality and consistency were limited. Long-term generalized monitoring has the least amount of evidence and requires further study. The concept of trade-offs between technology usefulness and acceptability is critical to consider, as older adults have a hierarchy of concerns. Implementing user-oriented frameworks can allow long-term and larger studies to be conducted to improve the evidence base for monitoring and increase the receptiveness of clinicians, policy makers, and end users.

## Introduction

### Background

Current health care systems are being pushed to use the capabilities of modern technology outside the hospital to increase efficiency and effectiveness of health delivery [1]. Transforming care processes by using digital platforms and remote monitoring may help address our increasingly older population and higher life expectancies [2]. Smart home and health monitoring technologies have been touted as the future for managing chronic diseases and allowing people to age in place [3-6].

With the technological advancements in the Internet of Things and the widespread use of machine learning and artificial intelligence, the application of smart home technology for aging in place has become more realistic and feasible. Numerous studies on technology development [3,7-10], clinical applicability [11-14], and user considerations [15-17] have been conducted to demonstrate that the technology is ready for mainstream use. There is a plethora of clinically evaluated activity and health recording devices readily available in the market, including wearables (eg, wrist bands, chest bands, and textiles) and ambient sensors (eg, motion sensors, cameras, and pressure sensors) [18-21]. However, the widespread adoption and development of health monitoring platforms remain limited for 2 reasons.

First, the evidence remains siloed within disease-specific reviews or secondary prevention, whether in heart failure [22,23], chronic obstructive pulmonary disease (COPD) [24], diabetes [25], or cardiometabolic health [26], making it difficult to compare effective monitoring models, delineate the overall evidence for home monitoring, and identify where gaps remain [27-29]. There is also a need to differentiate automated monitoring from user-based monitoring, which involves patients texting or phoning in their results.

Second, user and ethical concerns related to monitoring technologies remain major barriers to user adoption, particularly in research [30-33]. Much of the smart home research is focused on the technical development of devices rather than the reliability and usability of smart home systems [34]. Although the feasibility of smart home technology is high in most studies, the acceptability is a critical factor [35]. The benefits of the technology are touted without considering *if, how*, and *at what cost* a user may be willing to integrate the technology in their lives [17,36]. In addition, concerns related to data privacy and control, autonomy, and social connectivity are sometimes neglected when designing such systems [37-39].

### Objective

This review aimed to map out the literature on two major research questions: (1) what is the evidence for the effectiveness of home-based patient monitoring technologies for improving the health and well-being of older adults and (2) what are the ethical concerns that older adults have with home-based patient monitoring technologies, and what frameworks have been proposed to address these concerns? By addressing both questions, we aimed to provide a tool for researchers in this field to understand *what* needs to be studied and *how* to study them *while keeping in mind* ethical and user-centered practices.

In this study, we defined home health monitoring as the use of technology, omitting telecommunications, to monitor the health of users over time (ie, remotely) [40]. This could include using a variety of technologies, including wearables and ambient sensors, to track physiological parameters, activity levels, and routines or to facilitate rehabilitation and treatment [40].

The first part of this review outlined the clinical evidence for using home health monitoring through a scoping review of systematic reviews on home monitoring interventions. We highlighted the domains being researched; performed quality assessments; and determined whether the evidence is positive, neutral, or negative for home monitoring.

The second part of this review considered ethical concerns when researching and developing smart home monitoring technology and user-centered frameworks that address issues of acceptance and adoption. We outlined the ethical and user-centered frameworks that are available to improve these trials and described the current level of adoption of these frameworks in smart home monitoring.

## Methods

To answer the first research question, we conducted a scoping review by searching for systematic reviews following the PRISMA (Preferred Reporting Items for Systematic Reviews and Meta-Analyses) methodology [41,42]. The PRISMA checklist can be found in Multimedia Appendix 1.

### Data Sources and Search Strategy

Two researchers (AC and RC) conducted a probing search on MEDLINE for studies using smart homes related to older adults. We decided upon a summary of search terms with consultation with an interdisciplinary team of clinicians and engineers. We completed a systematic scoping search on Scopus, MEDLINE, Embase, and CINAHL in July 2021. The search focused on systematic reviews and meta-analyses using smart homes and remote monitoring of older adults (Table S1 in Multimedia Appendix 2).

### Study Screening and Inclusion and Exclusion Criteria

Three researchers (AC, RC, and KR) completed abstract screening and full-text screening. Inclusion criteria included systematic reviews written in English with >80% articles focused on older participants (aged >65 years) and published from 2010 to 2021. We excluded studies that focused on assistive technology, mobile health interventions, telephone- or videoconferencing-based interventions, mobile phones and apps, virtual reality, and robots because we focused on automated sensing technologies with clinician intervention. We also excluded studies in which >80% of the articles involved users texting or phoning in the results, rather than using automated monitoring systems. Narrative reviews and technical articles outlining the implementation of these technologies were excluded. We excluded studies that were not journal articles because our focus was on identifying gaps rather than estimating effect sizes.

### Data Extraction and Quality Assessment

We created an extraction table and completed extractions independently. The extracted items included population characteristics (number of articles, included diseases or disorders, and percentage of articles including older adults), study type (meta-analysis and quantitative or qualitative study inclusion), and monitoring methods (automated monitoring, mixed automated monitoring, and user-reported monitoring).

We extracted outcome measures, including physiological outcomes (vital signs and blood tests), symptoms or health events (falls, exacerbations, or mortality), health care use, cognitive decline, functional status, adherence to rehabilitation, and activity levels. Studies on human factors included user perspectives on the ethics of home monitoring, the acceptability and usability of devices, and changes in quality of life (QoL) or social connections through technology. We coded outcomes as positive, negative, mixed, or “not enough evidence” according to the author’s assessment and summarized in tables. Finally, we extracted the challenges that the author listed on health monitoring research, ranging from privacy and security concerns and technology acceptability to technical challenges and lack of clinical evidence. The challenges were coded based on what the authors reported. We developed a spreadsheet with a separate column for each challenge that each author would raise. If a new challenge was listed, it was added to a separate column. After we added all the articles and challenges, the 3 authors reduced the number of challenges by grouping together similar challenges by consensus.

Four authors (AC, RC, KR, and DB) conducted a quality appraisal using the AMSTAR-2 checklist and appraised independently after deciding upon critical, important, and unimportant categories. Categories rated as “Critical” included a comprehensive literature search strategy, describing included studies in adequate detail, and accounting for the risk of bias on evidence synthesis. Categories rated as “unimportant” in quality assessment included explaining the selection of study designs, providing a list of excluded studies, and reporting sources of funding for studies included in the review. All the other items were considered important. Studies with ≥2 “No” ratings on critical categories were considered critically low quality, whereas studies with one “No” rating were considered low quality. Moderate-quality studies had ≥2 “important” categories scored as “No,” whereas high-quality studies had <2 “important” categories scored as “No.” The entire list can be found in Multimedia Appendix 3.

### Ethics and Stakeholder Participation Review

To answer the second research question, we conducted 2 searches. The first search focused on the ethical challenges and concerns related to smart home technology. We conducted a systematic search from April to May 2021 using Scopus, Web of Science, and Dimensions AI. The search terms are listed in Table S2 in Multimedia Appendix 2. Once we collected all the papers, we screened the titles and abstracts to select the papers to be used for this review. Studies published before 2015 were excluded.

The second search focused on studies involving stakeholder participation while developing health care services and apps. We used Google Scholar to gather related chapters, journals, and articles with keywords (Multimedia Appendix 2). We excluded studies with health care frameworks that did not focus on user centeredness (ie, stakeholder involvement).

## Results

### Study Characteristics for Question 1: Evidence for Smart Home Technologies

**Figure 1:**
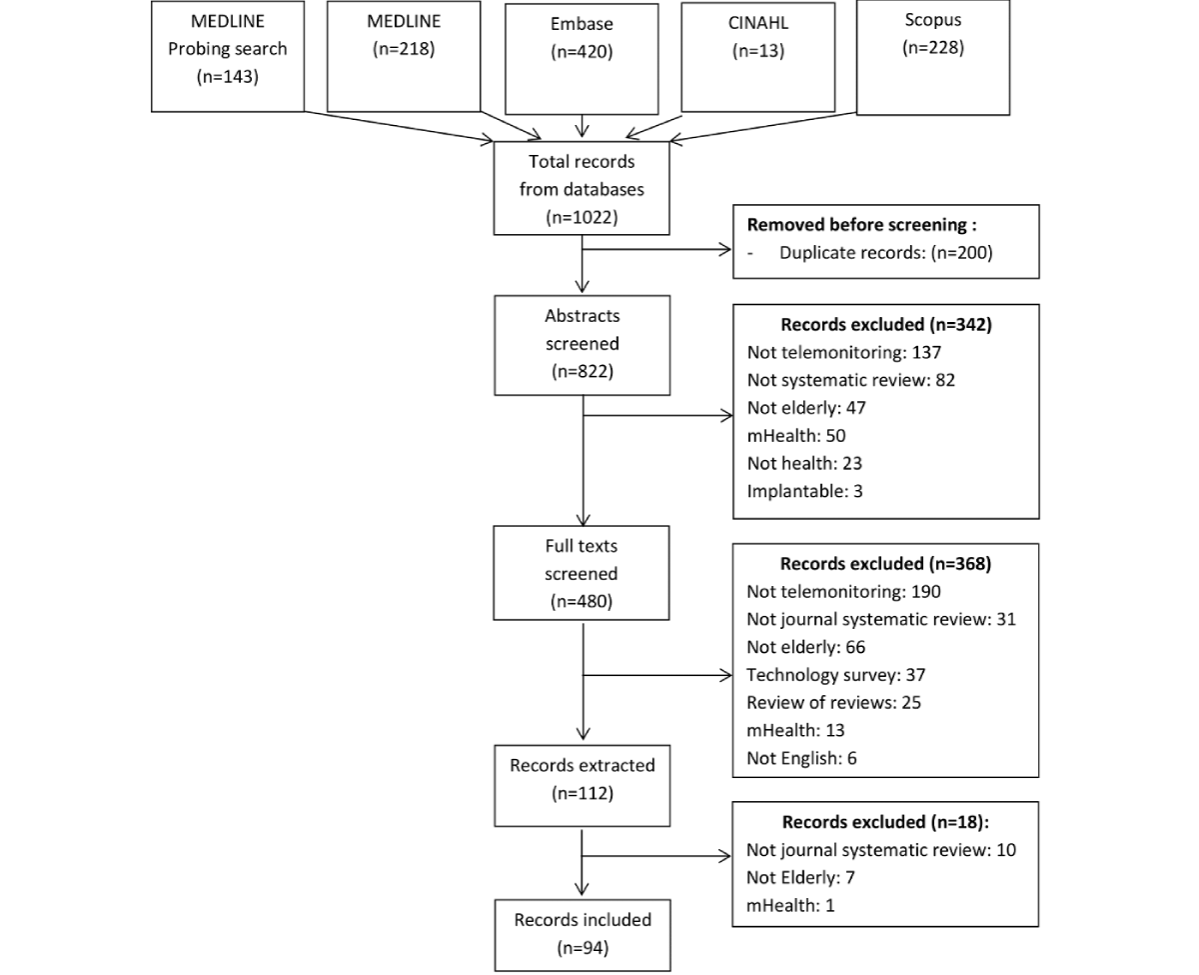
PRISMA (Preferred Reporting Items for Systematic Reviews and Meta-Analyses) flow diagram outlining the extraction process. mHealth: mobile health.

The search yielded 1022 articles, which was reduced to 822 after deduplication (Figure 1). Screening abstracts yielded 480 articles, whereas full-text and additional screening during extractions yielded 94 systematic reviews. Most articles were excluded because they did not focus on telemonitoring technologies (325/728, 44.6%), followed by articles that did not focus on older adult users (120/728, 16.5%) and that were not systematic reviews (104/728, 14.3%). The extraction procedures are presented in Multimedia Appendix 4.

**Figure 2:**
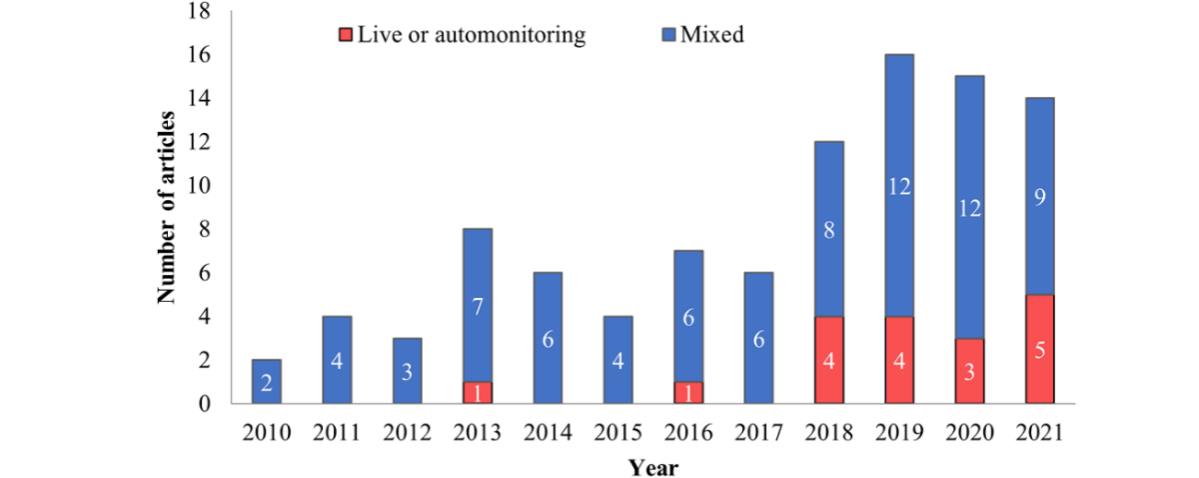
Number of systematic review articles published in each year which included only live or automated monitoring, or had mixed modes of monitoring.

Figure 2 shows an increasing trend for systematic reviews on home-based patient monitoring technologies, particularly starting in 2018. Live and automonitoring has only become more commonly reviewed since 2018. The number of systematic reviews showed an upward trend starting in 2018.

**Figure 3:**
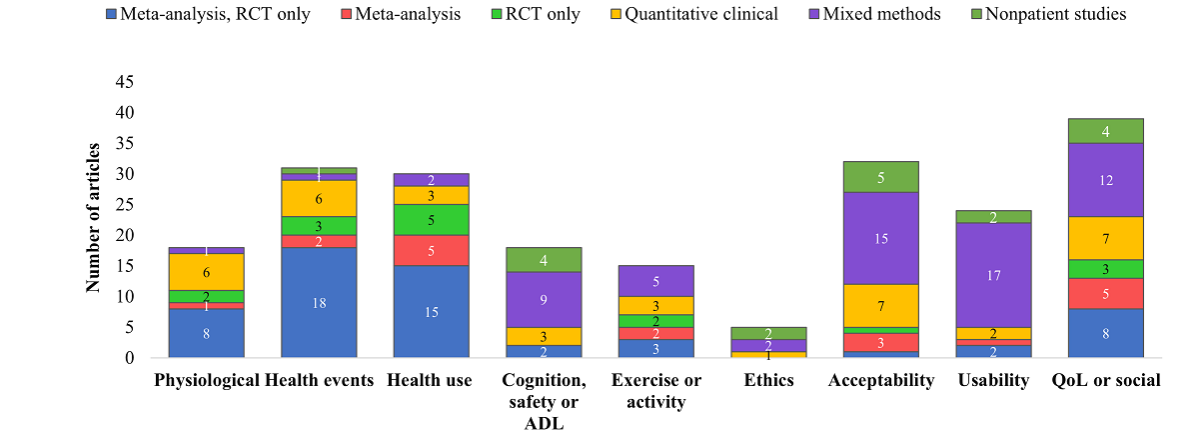
Number of articles according to outcome measures versus study design. ADL: activities of daily living; QoL: quality of life; RCT: randomized controlled trial.

Figure 3 displays the study designs used in each systematic review, organized according to the outcomes discussed. Many articles have presented >1 outcome category. Explanations of the outcomes are described in the methodology.

Meta-analyses were most common when reporting physiological evidence, health events, and health use (9/18, 50.0% for physiological; 18/31, 58.1% for health events; and 20/30, 66.7% for health use). Studies focusing on cognition, safety, and activities of daily living (ADLs) were mostly mixed methods or nonpatient studies (13/18, 72.2%), whereas studies focusing on exercise were mostly meta-analyses or quantitative clinical studies (10/15, 66.7%). Most systematic reviews on ethics, acceptability, and usability used mixed methods or nonpatient studies (4/5, 80% for ethics; 20/32, 62.5% for acceptability; and 19/24, 79.2% for usability), and no single study design dominated QoL or social-focused studies.

**Figure 4:**
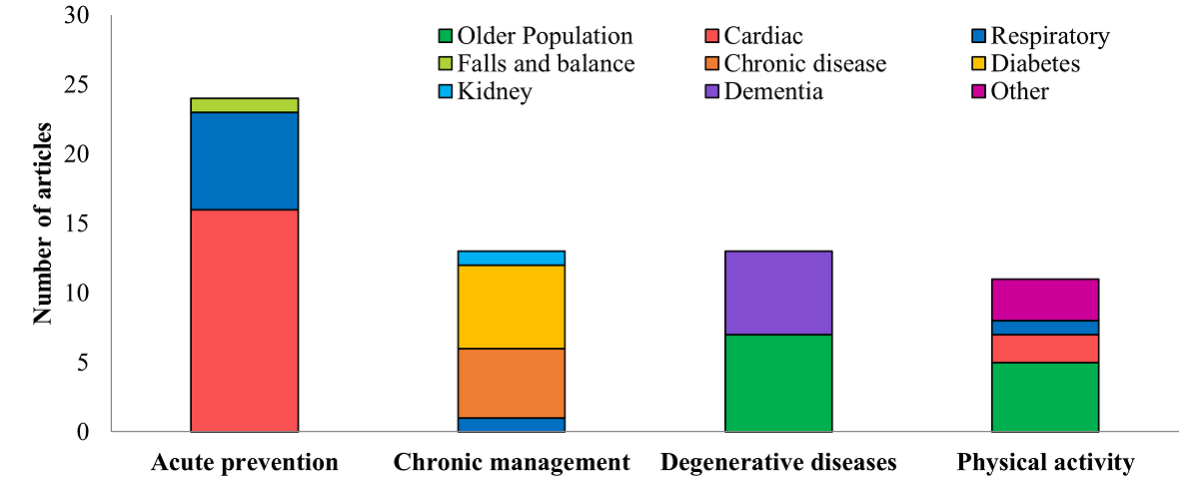
Disease processes organized according to the category of disease monitoring.

Figure 4 displays the disease processes studied according to the category of disease monitoring. Acute prevention studies focused on reducing heart failure and COPD exacerbations and fall prevention (24/61, 39.3% reviews). Chronic management studies focused on blood pressure reduction, blood glucose control, and metabolic disease management (13/61, 21.3% reviews). Home monitoring studies focused on monitoring aging and status of patients with dementia (13/61, 21.3% reviews). Studies on physical activity monitoring focused on rehabilitation or increasing physical activity in the older population (11/61, 18.0% reviews).

The challenges related to current evidence and implementation of home monitoring were also analyzed. Many studies listed multiple challenges, whereas 9.3% (9/94) studies listed no challenge. The most common challenges in the literature included a lack of strong clinical evidence for monitoring (510/94, 53.1%), poor descriptions of methodologies of how patients were monitored (27/94, 28.7%), and applicability to broader patient populations (10/94, 10.6%). On the implementation side related to human factors, the acceptance of technology (33/94, 35.1%), usability of devices (18/94, 19.1%), privacy concerns (17/94, 18%), cost-effectiveness (23/94, 24.2%), and safety concerns with devices (7/94, 7.6%) were listed. Technical challenges were not as commonly reported, although concerns with accuracy (10/94, 10.7%) and connectivity of devices (9/94, 9.7%) were more common than the others.

**Figure 5:**
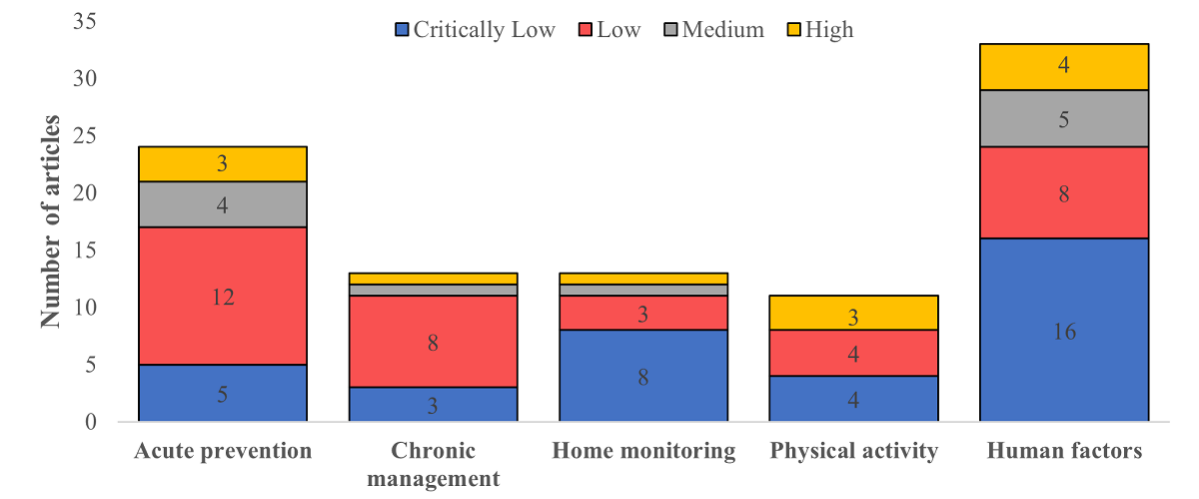
Number of articles listing challenges in smart home monitoring technology grouped by (top-to-bottom): evidence-based challenges, user-based challenges and technical challenges.

Figure 5 shows the AMSTAR-2 quality assessments organized according to the monitoring category. Most studies were either of critically low or low quality across all systematic reviews (36/94, 38.3%, and 35/94, 37.2%, respectively). Most studies did not discuss the impact of risk of bias on results (57/94, 60.6%), did not discuss heterogeneity (55/94, 58.5%), or did not include an explicit statement on following a protocol (50/94, 53.2%).

### Summary of Systematic Reviews on Evidence for Remote Monitoring

Table 1 reports the outcomes from articles that focused on clinical outcomes, as shown in Figure 4. The disease-specific details are presented in Multimedia Appendix 5. In total, 51 of 64 (80%) or more systematic reviews reported positive results across all categories. Chronic disease management and physical activity were among the categories with the largest number of systematic reviews with positive evidence. Evidence was most limited in managing degenerative diseases, health events, and use in rehabilitation interventions.

**Table 1:** Summary of evidence for home health monitoring according to category and outcome.

Outcomes		Reviews	Positive evidence	Negative or inconclusive evidence
**Acute prevention**				
	Health event (exacerbations, mortality, and falls)	22/24, (91%)	17/22 (77%) studies reported reduced exacerbations or mortality [43-57] High- or medium- quality: 4/22 (18%) studies reported reduced exacerbations [43,46,47,56]	5/22 (23%) studies showed no difference in mortality [58-62] 2/22 (9%) studies showed inconclusive results on mortality and exacerbations [63,64] High- or medium- quality: 3/22 (14%) studies showed no difference in mortality [58,59,61]
	Health use (hospitalizations and ER^a^ visits)	21/24, (88%)	16/21 (76%) studies reported reduced hospitalizations and ER visits [47-56,58-61,65,66] High- or medium- quality: 5/21 (24) studies reported reduced hospitalizations [45,47,56,59,61]	2/21 (10%) studies showed no change in hospitalization [43,57] 2/21 (19%) studies inconclusive on hospitalization [62,64,67,68] Increased hospitalization in 1/21 (5%) study on heart failure [63] High- or medium- quality: 1/21 (5%) study showed no change in hospitalizations in COPD^b^ [43]
**Chronic disease management**				
	Physiological outcomes (blood pressure, HbA1c^c^, and blood lipids)	10/14, (71%)	8/10 (80%) studies showed improved HbA1c [69-76] 5/10 (50%) showed improved blood pressure [69-71,73,77] 2/10 (20%) showed blood lipid reduction [69,76] High- or medium- quality: 1/10 (10%) study showed improved HbA1c [75]	1/10 (10%) study showed mixed evidence for change in HbA1c [77] 3/10 (30%) studies showed mixed evidence for blood pressure reduction [72,76,78] 1/10 (10%) study found no change in blood lipids [72] High- or medium- quality: 1/10 (10%) study showed mixed evidence for change in HbA1c [77]
	Health events (mortality, medical events, and pain)	4/14 (28%)	2/4 (50%) studies showed reduced mortality or adverse health events [69,70] No high- or medium-quality studies	1/4 (25%) study neutral for mortality for patients with chronic kidney disease [78] 1/4 (25%) study found medication adherence was mixed for varying medical conditions [79] No high- or medium-quality studies
	Health use for chronic disease (hospitalizations and ER visits)	5/14 (36%)	4/5 (80%) studies showed reduced admissions and ER visits [69,70,80] and treatment adherence [81] No high- or medium-quality studies	1/5 (20%) study neutral for hospitalizations [80] No high- or medium-quality studies
**Degenerative disease monitoring**				
	Function and independence	8/13 (61%)	3/8 (37%) studies showing that ADLs^d^ can be detected [82-84] 2/8 (25%) studies showing that QoL^e^ related to independence improved [85,86] High- or medium- quality: 1/8 (13%) study showing improved safety with assistive technology [87]	2/8 (25%) studies presented that technology is not mature enough to detect functional independence/ADLs [88,89]
	Cognitive status	5/13 (38%)	2/5 (40%) studies presented weak evidence for detecting cognitive impairment or agitation [90,91] High- or medium- quality studies: 1/5 (20%) study found high evidence for monitoring cognitive status and mental health [14]	2/5 (40%) studies presented that technology is not mature enough to detect cognitive status [92,93] High- or medium- quality studies: 1/5 (20%) study presented that overall technology readiness is low [14]
	Health use	2/13 (15%)	1/2 (50%) study found that monitoring cognitive status and mental health lowered hospital visits [14]	No reduced admission to care homes in 1/2 (50%) study [87]
**Physical activity**				
	Rehabilitation adherence or physical activity increase	11/11 (100%)	10/11 (90%) studies showed improved physical activity or adherence to rehabilitation [94-103] 1/11 (9%) study showed no change in rehabilitation outcomes compared with traditional rehabilitation [104] High- or medium- quality studies: improved physical activity in older adults, improved motor control in stroke, and improved pain in patients with arthritis [96,98,101]	—^f^

^a^ER: emergency room.

^b^COPD: chronic obstructive pulmonary disease.

^c^HbA1c: glycated hemoglobin.

^d^ADLs: activities of daily living.

^e^QoL: quality of life.

^f^Not available.

The first theme involved detecting acute events including exacerbations of heart failure, COPD, or falls (Table 1 and Table S1 in Multimedia Appendix 5). Most reviews reported on exacerbations of heart failure and COPD. Monitoring reduced hospitalizations for patients with COPD (5/7, 71.4% studies) and for patients with heart failure (10/14, 71.4% studies). Mortality was unchanged for COPD in a few reviews (3/7, 42.8%) but for most reviews for heart failure (11/14, 78.6%). Only 1 study had reviewed fall interventions in an older population [46]. No clinical studies have focused on atrial fibrillation, although devices showed high specificity and sensitivity in detecting atrial fibrillation.

Management of chronic diseases included managing diabetes, blood pressure, kidney function, or multiple diseases simultaneously (Table 1 and Table S2 in Multimedia Appendix 5). In most studies, home health monitoring helped to reduce hemoglobin A1c (8/10, 80%) and blood pressure (5/9, 55.6%), although the results were more mixed when only considering high- or medium-quality studies. In total, (2/4 (50%) of studies reported reduced mortality, reduced health events, and fewer hospitalizations when monitoring chronic cardiovascular diseases.

Smart home monitoring for ensuring safety of older adults with dementia or cognitive impairment had scarce evidence (Table 1 and Table S3 in Multimedia Appendix 5). Only 1/2 (50%) of studies found no reduction in admission to care homes for monitored patients [87]. In addition, 2/5 (40%) of studies noted progress in the ability to detect cognitive decline [90] and aggression [91]. Most studies found that technologies were not mature enough to detect activity changes or improve independence [88,89,92,105]. For monitoring older adults in general, % (3/N) of studies noted weak evidence for detecting changes in ADLs [82-84], and 2 studies showed improved QoL [85,86].

The fourth theme, rehabilitation adherence and encouraging an active lifestyle at home, included 11 studies (Table 1 and Table S4 in Multimedia Appendix 5). All studies showed positive outcomes for both disease-specific rehabilitation programs and for monitoring older adults in general. Monitoring improved adherence to cardiac rehabilitation, increased activity levels in patients with COPD, and improved motor control in patients after stroke [94-96]. Monitoring increased physical activity in older adults in general in 4 studies, and telerehabilitation with monitoring was found to be as effective as traditional rehabilitation [104]. Wearables helped to increase activity in patients with cancer, improve functioning in patients with arthritis, and improve QoL in postoperative patients [97-99].

### Summary of Systematic Reviews on Human Factors

As part of the scoping review, we included studies that focused on human factors such as acceptability of technology, ethical considerations, and costs. Although this was not the focus of question 1, we performed a basic analysis of the outcomes from these studies, recognizing the importance of human factors related to remote monitoring. Table 2 summarizes these 33 studies.

**Table 2:** Summary of studies focused on human factors (N=33).

Outcomes	Reviews, n (%)	Positive evidence	Negative or inconclusive evidence
Acceptability for managing chronic diseases	9 (27)	5 studies showing high acceptability of monitoring technology [106-110]	2 studies were inconclusive on monitoring acceptability [111,112] 2 studies were descriptive studies [17,113]
Acceptability of telerehabilitation	1 (3)	—^a^	One study was inconclusive on acceptability of monitoring in telerehabilitation [114]
Acceptability for home health monitoring	14 (42)	3 studies showing good acceptability of monitoring technologies [115-117]	2 studies showed inconclusive results on acceptability [105,118] 9 studies describing acceptability [36,119-126]
Costs	3 (9)	2 studies showed weak evidence that remote patient monitoring is cost-effective [127,128]	One study was inconclusive on cost-effectiveness of monitoring [129]
Ethics	3 (9)	—	3 descriptive studies on ethical frameworks for remote monitoring [37,39,130]
QoL^b^	3 (9)	1 study showed improved QoL with monitoring, though not by validated measures [32]	2 descriptive studies on QoL and social interaction with monitoring [131,132]

^a^Not available.

^b^QoL: quality of life.

Of the 24 studies on acceptability, 8/24 (33%) showed good acceptability of monitoring technologies in general. For chronic diseases, 5/9 (56%) showed good acceptability in monitoring for heart failure, COPD, and management of amyotrophic lateral sclerosis, whereas 2/9 (22%) studies on heart failure and COPD were inconclusive. Monitoring acceptability was less clear for generalized home health monitoring and rehabilitation for older adult patients with fatigue. Costs, ethical considerations, and QoL were inconclusive or were descriptive studies.

Although there appear to be positive results from specific diseases, there is a need to continue studying whether remote health monitoring is acceptable for the older adult population. Human factor considerations have been prominent in several studies and deserve closer inspection. Applying appropriate frameworks to the design and development of home health monitoring technology that address human factors such as users’ ethical concerns, like privacy and usability, need to be explored to ensure that users feel at ease while using and installing devices that monitor their lives. Therefore, the second part of our review focused on the ethical concerns that older adults have regarding home health monitoring technology and frameworks that have been suggested to address such concerns.

### Study Characteristics for the Ethics and User-Centered Frameworks

The second research question in this study was “what frameworks have been proposed to address the ethical concerns that stakeholders, specifically older adults, have toward home health monitoring?” To learn which frameworks are available to address the ethical issues that arise with home health monitoring, the ethical issues themselves must be uncovered and discussed in more detail.

To explore the ethical issues, we conducted a literature review of ethical challenges. An initial search of 132 papers published since 2015 was conducted, focusing on the ethical challenges and concerns regarding smart home technology. After screening titles and abstracts, 19 papers discussing the ethical challenges and concerns regarding smart home technology were identified. The most discussed concerns are listed in Table 3. The papers were a mix of systematic reviews, literature reviews, qualitative research (including focus groups and interviews), and mixed methods research (qualitative and quantitative research, many using surveys to obtain results).

**Table 3:** Summary of ethical concerns with smart home technology for the older population (N=19).

Outcomes	Reviews, n (%)	Methods used	Reason for concern
Privacy [31,33,37,133-142]	15 (79)	Literature review, mixed methods research, SR^a^, and qualitative research	Personal privacy concerns (watched and monitored) Informational privacy concerns (data sharing)
Control [33,37,133-137,139,140,143]	10 (53)	Literature review, mixed methods research, SR, and qualitative research	Fear of losing control and desire to make decisions about the technology (use, on or off, placement, and data collection)
Social concerns [33,37,133,135,137,140-142,144]	9 (47)	Literature review, mixed methods research, SR, and qualitative research	Desire for face-to-face communication and fear of losing in-person interaction
Autonomy [31,33,37,133-135,137,140,143]	9 (47)	Literature review, mixed methods research, SR, and qualitative research	Very important for older adults Do not want to be a burden on others Fear of being dependent
Stereotypes and stigmatization [31,33,37,134,138]	5 (26)	Literature review, mixed methods research, and qualitative research	Fear of judgment and its consequences and stereotypes

^a^SR: systematic review.

### Ethical Challenges and Concerns

#### Overview

The first half of this review made it apparent that home monitoring of older adults is a useful intervention. However, many studies have highlighted the ethical challenges and impacts on user acceptance and adoption that arise when using monitoring technology.

When home monitoring technology for aging in place is used appropriately, it can improve QoL, maintain health and wellness of older adults, and support other stakeholders [37]. Such technology may allow increased autonomy and independence in older adults while providing additional support for family members or health care professionals [133]. However, stakeholders and researchers have raised many ethical concerns regarding the design, development, and deployment of home monitoring technologies. This section expands on the most discussed ethical questions and concerns regarding home health monitoring technology, as outlined in Table 3. By understanding these concerns, solutions may be discovered to better design and implement home monitoring technology for older adults and other critical stakeholders [37].

#### Privacy

Privacy is one of the most critical factors affecting older adults’ willingness to participate in and use smart home technology [31,33,37,133-138,143]. Privacy can be classified into 2 types: physical and informational. Physical privacy relates to the degree to which a person or their personal space is physically accessible [136]. The home is a refuge for privacy and intimacy [139], so it is understandable that some users of home monitoring technology may feel discomfort or apprehension toward any device that can watch them, like an “invisible person” in the room [134,135,137,143]. Any technology that impinges on this refuge will have lower acceptance rates [31,33]. Informational privacy refers to the desire of a person to control the sharing of personal information with others [136]. Informational privacy can be violated when information is used against the wishes of the stakeholder [135]. As home monitoring devices may store and transmit intimate personal data, many older adults, their family members, and health care professionals have reservations about data collection policies including types of data collected, use of the data, and access levels [31,134,139]. Interestingly, the results of a study focused on designing home monitoring technology found that although information privacy was a priority for engineers and designers, physical privacy implications were not considered [143]. This highlighted the need to understand both the user’s and designer’s perspectives and to think broadly about privacy.

#### Control

Control has multiple meanings including controlling device data, settings, and who makes decisions about the device and the data it collects [31,37,134]. Older adults desire to maintain control of their lives and surroundings for as long as possible, with many seeing value in home monitoring, but more as a last resort or to be used later in life [31,134,138-140]. Older adults want to control whether and how to use the technology, when it is turned on or off, where it is placed, and with whom the collected data are shared [37,134]. From the designer’s perspective, Birchley et al [143] found that a common trend was to give end users the responsibility to decide what the technology did and how much control it had in their lives. However, it was pointed out that many of the ethical challenges regarding the design and implementation of technology, such as an opt-out feature for data sharing, were addressed in the early phases of design, which placed the responsibility of ethical design with the engineers and designers, not the end users [143].

#### Social Concerns

As shown in Table 3, social interaction is a growing ethical concern. Many older adults strongly indicate that technology should not replace human contact but should foster and promote human communication and support [140,141]. An increase in assistive technology could mean a decrease in human care and human contact for older adults [37,140,141,145]. Older adults, family members, and health care professionals insist that face-to-face interactions should not be systematized or replaced by technology [37,145]. For many older adults, visits from health care workers are often the only human contact they receive on a day-to-day basis, making this interaction critical to their health and well-being [37,140].

#### Stereotypes and Stigmatization

Stereotypes of “oldness” are often depicted as a time of ill health, dependency, and loneliness [31,33,37]. However, older adults want to be perceived as strong, capable, and independent individuals [31,134]. Any device that projects negative aging stereotypes is likely to be rejected by older adults, even if the device is helpful [31]. Of the older adults interviewed by Astell et al [31], many expressed fears of being judged or discriminated against if they used devices that would stigmatize them as being different, incompetent, or lonely. Participants were more likely to decline social events than to use devices that could incur judgment [31]. Home monitoring devices for older adults must not reflect stereotypes or stigmatizations but should augment how older adults view themselves as independent, competent, and self-reliant users.

#### Autonomy

Autonomy for smart home technology means “the assistive technology developed for elderly care must not interfere with the will of the person it is designed to care for” [133]. Older adults strive to maintain independence and personal autonomy to avoid burdening their loved ones or society [31,37,140,141]. Therefore, devices that enable or prolong independent performance in meaningful activities are met with great enthusiasm, although some still hold reservations regarding how home health monitoring could affect their autonomy and independence [31,37]. Some older adults expressed concerns about becoming overreliant on the devices [146], whereas others did not want technology to complete a task without them [140]. Nevertheless, older adults overwhelmingly agreed that if home health monitoring technologies could preserve their autonomy and accommodate their preferences, using the technology was preferable to moving into a nursing home [140]. Table 3 summarizes the most discussed ethical concerns along with how often they were mentioned in the collected papers.

Although many older adults admit that they see value in home health technology, they also have wide-ranging reservations about it. The ability to anticipate, address, and respond to ethical challenges and concerns is critical for future development and adoption for stakeholders. Communication between all involved stakeholders must occur to better understand the attitudes, concerns, and demands of those who are most impacted by the technology [31,134,147,148].

### Impacts on User Acceptance and Adoption

#### Overview

In addition to the ethical concerns and challenges for home health monitoring technology discussed, many studies have also examined how ethical challenges and concerns impact user acceptance and adoption of smart home monitoring devices [31,33,37,134,138,142,149-155]. Table 4 highlights the most discussed user aspects found in the literature that influence user acceptance and adoption of home health monitoring technology.

**Table 4:** Summary of older adult population’s user aspects concerning smart home technology (N=19).

Concern	Papers, n (%)	Methods used	Feedback from users
User thoughts and feelings (eg, attitudes, preferences, and knowledge) [31,37,133,134,138,142,145,147-149]	10 (53)	Literature review, mixed methods research, and qualitative research	Positive and negative views on aging change acceptance level Limited knowledge on technology but older adults are willing to learn and desire customizable technology
User acceptance [31,33,37,134-137,142,143]	9 (47)	Literature review, mixed methods approach, SR^a^, and qualitative research	Many factors influence acceptance and excitement for smart home technology but do not think they need it Technology must respect certain values
Usability [37,134,142,144,145]	6 (32)	Literature review, mixed methods research, and qualitative research	Technology should be easy to understand and use
Usefulness [37,133,134,142,145,149]	6 (32)	Literature review, mixed methods research, and qualitative research	Technology must have a purpose Older adults want feedback from the technology to know what it is doing
User adoption or abandonment [31,33,37,142]	4 (21)	Literature review, mixed methods research, and SR	To promote adoption, stakeholders need to be included in discussions around developing technology

^a^SR: systematic review.

Often, health care tools are designed without considering the values and characteristics of the intended users and other key stakeholders, their literacy levels, or their information goals and preferences. This can result in the technology suffering “social failure modes” [156], ultimately leading to the abandonment of the technology and the need to redesign it [150]. Web-based medical platforms are one example, having low adoptability or acceptance rates [1], potentially because of inadequate user involvement, especially in the early development phases [151-153].

#### User Acceptance, Usefulness, and Usability

User acceptance is not only based on excitement with technology [31,37] but also on appearance, values and principles, situation, usability, opinions of other direct stakeholders [134], and especially attitude [31,37,134,142]. Older adults who have negative views on aging and associate it with traits such as “illness,” “loneliness,” or “dependency” tend to neglect assistive technology they believe reflects those ideals, whereas those who see aging as positive are more inclined to accept assistive technology and integrate it into their daily lives [31,134]. If end users see no benefit in using an assistive device, there is little chance of acceptance [37,149]. A device that is difficult to operate or understand can lead to frustration and lack of confidence [138,142,149]. Usefulness and usability also tie into developing technology that respects and accommodates end user’s values. If older adults believe that their values are being threatened without explanation, they will likely refuse to accept the technology, whereas they will be more likely to adopt it if those values are upheld [33,134]. All the factors discussed need to be weighed to determine the value of the technology. Tools should be tailored to the users to increase the usability and utility of the technology [154]. The transition from “doing for” users to “doing with” users requires considerable adjustments to be made in both attitude and practice [155].

#### User Adoption or Abandonment

Beyond the acceptability of technology is the adoption of these devices by end users. Chung et al [37] noted that for aging in place technology to be truly adopted by older adults, devices should address older adults’ values, self-perceptions, and ethical issues at the intersection of aging, technology, and the home environment. Device abandonment is a common reality, whether it is because the device impedes a user’s independence or because of the fear of judgment from their peers [31]. Careful decisions must be made throughout the development process to ensure that the final deliverable is something that is beneficial to the end user and aligns with how the end user sees themselves.

### Frameworks for User-Centered Design

Health care tools are often designed without considering the intended users, their literacy levels, information goals, and preferences, which results in user dissatisfaction, leading to the abandonment of the technology and eventually the need to redesign it [150]. Despite constant advancements, web-based medical platforms have low adoptability or acceptance rates [1]. A reason for this is inadequate user involvement, especially in the early development phases [151-153]. We should aim to tailor the technology to the stakeholders’ needs and requirements to increase their usability and utility [154]. User-centered design (UCD) is a framework in which the requirements of stakeholders (including end users) are considered extensively at every stage of the product’s development and design [157]. Table 5 summarizes the various user-centered frameworks developed and adopted in various health care settings based on systematic reviews and individual articles. Moreover, the use of UCD-based evaluation instruments, such as the 11-item measure “UCD-11,” developed by Witteman et al [154] to quantitatively determine the user centeredness of the design and development of health care tools will ensure production of reliable and valid constructs.

**Table 5:** User-centered frameworks in medical design.

Framework	Description
3-phase framework: narrative, metaphorical, and structured [2]	Narrative phase gathers patients’ every-day stories to develop design goals in the metaphorical phase, followed by building technological prototypes in the structured phase
3-phase iterative framework in repeated cycles [153]	Identifying users and understanding their environments, developing a prototype, and refining the prototype through observations and feedback
Design thinking framework (5 modes executed iteratively, either sequentially or parallelly) [158]	Understanding user perspective in the empathize mode, synthesizing user feedback into identifiable needs in the define mode, identifying diverse solutions to the problem statement in the ideate mode, constructing basic but actual representations of the ideas in the prototype mode, and simulating the developed prototypes in context of the problem and refining or improving them in the test mode
Information systems research framework (3-phase framework) in combination with the 5 modes of the design thinking framework [158,159]	The relevance cycle (understanding the end user environment) combined with the empathize and define modes, the design cycle (creating and assessing objects related to the problem) combined with the ideate and prototype modes, and the rigor cycle (appending findings from evaluation in existing knowledge base) combined with the test mode
The website development model for consumers [150]	Analyzing user requirements; evaluating environments; defining website functions, constraints, visuals and structure; and testing with small-scale user groups
Wearable device design framework (3-level, top-down) [160]	Level 0: classifying design requirements into physical, cognitive, and emotional ergonomics Level 1: generalizing the requirements into comfort, durability, safety, reliability, usability, engagement, and aesthetics Level 2: specifying level 1 requirements to enable measurement, either quantitative or qualitative
The eHix^a^ framework [161,162]	Consists of a 20-cell matrix constructed from 4 business model domains (Service, Technology, Organization, and Financial), each having 5 phases of innovation (Inventory—new ideas explored and user needs and requirements identified, Design and Development—construction of prototype application or technology, Experimental—testing the developed prototype in a laboratory setting, Pilot—testing the prototype in daily life scenarios and providing feedback, and Implementation—the finished prototype is deployed). Each cell lays out the required steps and instructions for a particular phase in each domain

^a^eHix: eHealth innovation matrix.

## Discussion

This paper had 2 aims: to present the evidence for home health monitoring through a scoping review and to provide the ethical, user-centered considerations and potential frameworks that can be adopted for developing user-centered health care platforms and personalized support systems. The implementation of remote monitoring requires both a strong backing of the evidence for monitoring to demonstrate its benefits, while maintaining a focus on the ethical and user implications of remote monitoring.

### Principal Findings

#### Evidence for Home Health Monitoring

The first half of this paper presented the evidence related to home health monitoring. We found numerous systematic reviews, with the majority showing positive evidence in monitoring for acute exacerbations of COPD and heart failure, improving blood pressure or diabetes markers, and increasing physical activity levels. When only high- or medium-quality articles were considered, monitoring for COPD and heart failure had the strongest evidence, diabetes had mixed results, and rehabilitation and physical activity had positive results.

Compared to a previous review of systematic reviews on telemedicine in 2010 [163], advancement can be seen in managing heart failure, COPD and for improving HbA1c levels. This result corresponds with the current literature, with a Cochrane review showing positive results in COPD and heart failure [164], another 2 showing improved diabetes management [165,166], and another showing equivalent outcomes comparing telerehabilitation with conventional rehabilitation, although monitoring was not the focus of this review [167].

#### Ethical Concerns and User-Centered Frameworks

Usability and ethical acceptability are critical for fostering adoption among older adults. The concept of a “trade-off” was key—where multiple factors cannot be attained at the same time, stemming from value tensions between older adults and technology [168]. The value tension between autonomy and privacy was common, where older adults were against being monitored by technology but were willing to relinquish some privacy if they could stay at home longer [37,134,136,139-141,143,149]. Other examples of trade-offs included safety and privacy [134,144], utility and privacy [33,139], and social interaction and privacy [149].

Moreover, end users find disparity between their requirements and the eHealth solutions provided to solve their problems [152]. Although developers may not approach the problem from the users’ perspective [150,152], UCD in eHealth comes with additional practical challenges, including ambiguity regarding the number of iterative cycles to be conducted, time and cost constraints associated with the process, difficulty in overcoming designer bias, and difficulties in establishing multidisciplinary collaboration [169]. To mitigate such challenges, researchers and developers are recommended to refer to established, flexible, reliable, and valid UCD frameworks with respect to the problem statement and the target population.

### Limitations and Gaps in the Evidence

The literature on home monitoring is limited. First, the study quality was limited to secondary prevention in heart failure and COPD from 7 high- or medium-quality studies and encouraging rehabilitation and physical activity in 3 studies. Most systematic reviews were of low or critically low quality. We have presented the results of a subgroup analysis of only high- and medium-quality studies to mitigate our own risk of bias. Higher-quality systematic reviews are critical for chronic disease management and generalized monitoring.

Second, the older adult population is not always the focus of reviews. We found only 1 fall detection study in older adults using monitoring devices. A technology survey of fall detection systems found that only 4 out of 57 studies even included older adults in their study [170]. In the screening process, >120 reviews were excluded, as they did not focus on adults aged >65 years. It is important to differentiate these populations, as the acceptability of technology and challenges of multiple morbidities may change the effectiveness of monitoring.

Third, the evidence was sparse for monitoring older adults in general or patients, with most studies suggesting that the technology is not yet mature enough to detect ADLs or cognitive decline [89,92,93]. Most acceptability studies have focused on the domain of general home health monitoring, with the majority being descriptive or showing mixed acceptability. The question of whether it is worthwhile to monitor older adults in general or those with dementia remains unanswered. In addition, telemonitoring and telemedicine are often not differentiated, making it difficult to differentiate the benefits of clinician support from devices and technological support.

From an evidence-focused perspective, monitoring is effective for specific diseases, but challenges remain in researching generalized monitoring for older adults. Clinical outcomes are difficult to measure because objective physiological markers of aging and health utilization need to be measured across years to determine effectiveness. Second, technical obstacles remain in acquiring data from multiple sensors, synchronizing outputs in real time, storing the data, and performing data analytics to detect anomalies or track wellness, all of which can be time consuming and expensive. Finally, the drivers of long-term monitoring are older adults or families focused on the personal benefits of aging in place. Outcomes focused on improving function and independence and older adults’ sense of security and identity may not draw as much broad attention or funding compared with long-term clinical effectiveness and cost-effectiveness studies. Considering the promising results of remote monitoring in specific apps, it is worth researching broader generalized monitoring to improve clinical outcomes.

### Limitations of This Review

The main limitation of our review was that we did not have a 2-person validation for screenings and extractions. This may have introduced a bias in the included studies and the information extracted. To mitigate the validation of screenings and extractions, we used common inclusion and exclusion criteria and communicated with team members regularly on questionable papers. In addition, our search was limited to clinical databases to focus on clinical effectiveness rather than technological developments; this may have biased our results related to human factors and monitoring. Moreover, it is possible that multiple studies were included in multiple reviews, which may have biased the results. However, this cannot be mitigated. Finally, we identified gaps in the data but were unable to quantify the effect sizes.

Regarding the ethics- and user-centered framework portion of the review, we did not follow a scoping review methodology, as we found that the qualitative nature of the findings could be summarized with a narrative review. However, we may have missed some trade-offs regarding the acceptability of monitoring technologies.

### Future Work

An interesting area for future research is the extraction of what acceptability tools were used in each study. For the second half of this review, we did not provide an exhaustive list of the ethical concerns that end users have regarding home health monitoring technology. We also limited our study to older adults and did not consider family members; health care professionals; and other stakeholders such as engineers, computer scientists, and designers. More value tensions will arise and need to be addressed with the involvement of more stakeholders.

### Conclusions

This scoping review provided a summary of the clinical evidence for monitoring older adults in their homes, ethical implications, and user-oriented frameworks found in the literature. Overall, there is promising evidence for monitoring specific diseases and for rehabilitation support, but generalized monitoring for older adults, including cognitive and physical decline, has not been well researched. More clinical research is required for the long-term monitoring of aging in place to provide evidence for its use. To conduct these future studies, we performed a review of important ethical and user considerations and existing user-centered frameworks that must be considered when conducting these monitoring studies. This study demonstrated the need to develop technology with stakeholders rather than for stakeholders to build the evidence for home health monitoring. User-centered frameworks allow stakeholders’ ethical concerns to be addressed and open iterative design opportunities to improve adoption. In developing a system that achieves ethical and UCD, researchers can collect long-term, meaningful data to demonstrate the efficacy of home health monitoring systems for aging in place.

## Appendix

Multimedia Appendix 1 PRISMA (Preferred Reporting Items for Systematic Reviews and Meta-Analyses) checklist for evidence for remote monitoring.

Multimedia Appendix 2 Search strategy on evidence for remote monitoring.

Multimedia Appendix 3 Quality assessments for evidence on remote monitoring.

Multimedia Appendix 4 Extraction tables for evidence on remote monitoring.

Multimedia Appendix 5 Summary of evidence for remote monitoring.

## Abbreviations

ADL: activity of daily living
COPD: chronic obstructive pulmonary disease
PRISMA: Preferred Reporting Items for Systematic Reviews and Meta-Analyses
QoL: quality of life
UCD: user-centered design

## Data Availability

We have included the search strings in Multimedia Appendix 2, quality assessments in Multimedia Appendix 3, the extracted articles in Multimedia Appendix 4, and more detailed summaries of the articles extracted in Multimedia Appendix 5 to provide transparency on extractions.
