# Supplementary material for: Evidence and User Considerations of Home Health Monitoring for Older Adults: Scoping Review
Source: JMIR Aging. 2022 Nov 28;5(4):e40079. doi: 10.2196/40079 (PMC9745651; doi:10.2196/40079)
Supplement: Multimedia Appendix 2 [file aging_v5i4e40079_app2.docx]

# Search Strategy

The list of key words was developed after an exploratory search and discussion between authors. Since we were only including systematic reviews, we kept our search strategy relatively broad, to only include population in the PICO question, but we wanted to target diseases that would be particularly more common in older adults.

MEDLINE Search:

1 elderly or senior or older adult

2 cardiac or heart or pulmonary or lung or respiratory or renal or kidney or fall* or arthritis or frail* or diabetes or blood pressure or dementia or mild cognitive impairment or Parkinson or amyotrophic lateral sclerosis or exercise or physical activity or telerehabilitation or rehabilitation

3 smart home* or telemedicine or remote monitoring or ioT or home health monitoring or remote patient monitoring

4 1 or 2

5 3 and 4 and 5

6 limit 5 to “all aged (65 and over)” and (meta analysis or “systematic review”)

Table B.1. List of search terms and topics

| **Population Search Terms** | **Smart Home Terms** | **Acute Event Detection** | **Chronic Disease Management** | **Monitoring for Degenerative Disease** | **Encouraging Active, Healthy Lifestyles** |  |
| --- | --- | --- | --- | --- | --- | --- |
| Elderly  Aged  Senior | Smart Homes  Tele-health  Tele-medicine  Remote Monitoring  IoT (Internet of Things) | Falls and Frailty  Heart Failure, Cardiac  Respiratory, Pulmonary, COPD | Blood Pressure  Diabetes  Arthritis  Kidney, Renal, Chronic Kidney Disease (CKD) | Dementia  Mild Cognitive Impairment  Parkinson’s  Amyolateral Sclerosis  Ambient Assisted Living, Activities of Daily Living (ADL) | Exercise  Physical Activity  Rehabilitation  Tele-rehabilitation | |

Table B.2. Search terms and topics for the second research question

| **Population Search Terms** | **Smart Home Terms** | **Ethical Implications** | **User-Centred Frameworks** |
| --- | --- | --- | --- |
| Elderly  Senior  Older Adult | Smart Homes,  Remote Monitoring,  Ambient Assisted Living (AAL) Technology,  Ageing in Place,  Intelligent Assistive Technology | Ethics  Ethical  Implications  Ethical Concerns  Ethical Challenges | User-Centred  Design,  User  Involvement,  Patient Participation,  Stakeholder Engagement |
