# Supplementary material for: Evidence and User Considerations of Home Health Monitoring for Older Adults: Scoping Review
Source: JMIR Aging. 2022 Nov 28;5(4):e40079. doi: 10.2196/40079 (PMC9745651; doi:10.2196/40079)
Supplement: Multimedia Appendix 5 [file aging_v5i4e40079_app5.docx]

# Multimedia Appendix: Summary of Evidence Tables

Table E.1. Summary of Evidence for Smart Homes and Acute Event Detection/Prevention

| Disease/issue | | # Total Reviews | Positive Evidence | Negative, Inconclusive Evidence |
| --- | --- | --- | --- | --- |
| COPD [1–7] | | 7/24 | Fewer ER visits and hospitalizations in 5 studies (71.4%) [2,3,5–7], Fewer exacerbations in two studies (28.6%) [1,4] | Not enough evidence on hospitalizations in 1 study (14.3%) [1], no change in mortality in 4 studies (57.1%) [3,5–7] |
| Heart Failure [8–23] | | 14/24 | Improved mortality in 11 studies (78.6%) [9–16,18,19,22], reduced hospitalizations in 10 studies (71.4%) [9–16,18,19]. | Inconclusive for health utilization in 4 studies (25.5%) [8,17,20,23] , 1 study reported no change [22], 1 study showed more health utilization [21]  One study inconclusive on mortality [21], one study inconclusive on exacerbations [23], one study reported no change on mortality with monitoring [17]. |
| Falls [24] | | 1/24 | Falls prevention interventions can reduce falls, not necessarily monitoring interventions [24]. |  |
| **Other Diseases** | |  |  |  |
|  | Cardiopulmonary Events [25] | 1/24 | Reduced hospitalization in cardiovascular disease and COPD [25] |  |
|  | Atrial Fibrillation [26] | 1/24 | Increased detection of atrial fibrillation [26] |  |

Table E.2. Summary of Evidence for Smart Homes and Managing Chronic Diseases

| Disease | | # Total Reviews | Positive Evidence | Negative, Inconclusive Evidence |
| --- | --- | --- | --- | --- |
| Diabetes [27–32] | | 6/13 | Reduced HbA1c and improved blood pressure in three studies and HbA1c alone in one study (66.7%) [27–30] | Inconclusive results in two studies (33.3%) [31,32] |
| Multiple Chronic Diseases [33–37] | | 5/13 | Reduced HbA1c in two studies (40%) [33,34], reduced hospitalizations and ER visits in three studies (60.0%) [33,34,36] | Inconclusive glycemic control in one study [37]. |
| Other: | |  |  |  |
|  | Chronic Kidney Disease [38] | 1/13 |  | Blood pressure and mortality unchanged [38]. |
|  | Sleep Apnea [39] | 1/13 | Improved treatment adherence in sleep apnea [39]. |  |

Table E.3. Summary of Evidence for Monitoring in Degenerative Processes.

| **Disease** | **# Reviews** | **Positive Evidence** | **Negative, Inconclusive Evidence** |
| --- | --- | --- | --- |
| Dementia [40–45] | 6/13 | One study found weak evidence for detecting cognitive impairment/symptoms using sensors [41]  One found limited evidence that agitation and aggression can be detected [42]  One found safety improved with assistive technology [40] | Two study found most studies to detect activity/symptoms of MCI were not mature (33%) [43,45]  One study inconclusive on the ability for monitoring to reduce care home admission [40].  One study found no evidence that technology improves independence/reminders, safety, and lives of people with dementia [44] |
| Other [46–52] | 7/13 | Three studies found evidence to detect ADLs or changes in ADLs [48–50]  Two studies found general improved QoL[51,52]  One study found monitoring cognitive status and mental health improved outcomes and lowered hospital visits [46] | Two study found technology is still immature in detecting ADLs or in general [46,47] |

Table E.4. Summary of Monitoring for Healthy Lifestyles and Rehabilitation

| Disease | # Reviews | Positive Evidence | Negative/  Inconclusive |
| --- | --- | --- | --- |
| Rehabilitation Programs [53–56] | 4/11 | One study showed positive adherence to cardiac rehab programs and greater physical activity level. [53]  One study showed positive effect on hospitalizations and activity levels for COPD [54]  One study showed improved motor control post-stroke, though high heterogeneity [55]  One study showed equivalent outcomes with telerehabilitation with reduced resource utilization [56] |  |
| Elderly in General [57–60] | 4/11 | Four studies (80.0%) found adherence to physical activity and gait improved using technology [57–60] |  |
| Other: Cancer (1) [61], Post-operation (1) [62], Arthritis (1) [63] | 3/11 | Increased physical activity in cancer patients [61], post-op patients have improved QoL [62], one study showed improved physical functioning in arthritis [63] |  |
